# The origin of gastric cancer stem cells and their effects on gastric cancer: Novel therapeutic targets for gastric cancer

**DOI:** 10.3389/fonc.2022.960539

**Published:** 2022-09-15

**Authors:** Ying Yang, Wen-Jian Meng, Zi-Qiang Wang

**Affiliations:** Colorectal Cancer Center, Department of General Surgery, West China Hospital, Sichuan University, Chengdu, China

**Keywords:** gastric cancer, gastric cancer stem cells (GCSCs), targeted therapy, drug resistance, relapse

## Abstract

Gastric cancer (GC) is one of the most prevalent malignancies and the most common causes of cancer-related mortality worldwide. Furthermore, the prognosis of advanced GC remains poor even after surgery combined with chemoradiotherapy. As a small group of cells with unlimited differentiation and self-renewal ability in GC, accumulating evidence shows that GC stem cells (GCSCs) are closely associated with the refractory characteristics of GC, such as drug resistance, recurrence, and metastasis. With the extensive development of research on GCSCs, GCSCs seem to be promising therapeutic targets for GC. However, the relationship between GCSCs and GC is profound and intricate, and its mechanism of action is still under exploration. In this review, we elaborate on the source and key concepts of GCSCs, systematically summarize the role of GCSCs in GC and their underlying mechanisms. Finally, we review the latest information available on the treatment of GC by targeting GCSCs. Thus, this article may provide a theoretical basis for the future development of the novel targets based on GCSCs for the treatment of GC.

## 1 Introduction

Globally, gastric cancer (GC) is the fourth-leading cause of cancer-related deaths worldwide and was responsible for an estimated 769,000 deaths in 2020 (1). Some risk factors for GC, such as Helicobacter pylori (HP) infection, have also been confirmed by most studies. In fact, there are significant differences in risk factors, carcinogenicity and epidemiological patterns between GC located in cardia and GC located in non-cardia regions (1). A major cause of non-cardia GC is chronic HP infection, and this bacterium is responsible for almost all non-cardia GC (2, 3). In addition, poor dietary habits, such as drinking alcohol, smoking, eating salted foods, low intake of fruits and a high-fat diet, have also been linked to an increased risk of non-cardia GC (4, 5). The risk factors for gastric cardia cancer are similar to those for esophageal adenocarcinoma, such as acid reflux disease lesions, compared to non-cardia GC (1). However, the exact pathogenesis of GC remains unknown.

In recent decades, with the rapid development of cancer stem cells (CSCs) research, it is becoming appreciated that CSCs (also known as tumor-initiating cells) with high oncogenicity are the source of the development, recurrence, metastasis and drug resistance of tumors (including GC) (6–9). Clinically, decision making for GC treatment is usually based on the tumor TNM stage and overall health status of the patients. For patients with resectable GC without metastasis, multimodality therapy can improve the prognosis. However, despite continuous improvement in treatment regimens, the outcome of patients with advanced GC remains poor (10). Therefore, it is particularly important to develop new and more effective therapies for the advanced GC. CSCs are a small fraction of tumor cells with the characteristics of self-renewal, clonal tumorigenesis potential, differentiation and long-term repopulation (11, 12). They are closely related to tumor heterogeneity and play a key role in clinical phenomena such as recurrence after the initial success of chemoradiotherapy, tumor dormancy, tumor metastasis and drug resistance (13–15). Similarly, gastric cancer stem cells (GCSCs) are responsible for GC progression, recurrence, metastasis, and drug resistance (16, 17). However, the origin of GCSCs and the exact mechanism by which GCSCs may promote the progression, recurrence, metastasis and drug resistance of GC remain unclear. Therefore, this paper will summarize the key concepts, sources and characteristics of GCSCs and discuss the role of GCSCs in GC and its underlying mechanisms. It also provides up-to-date information about targeting GCSCs to treat GC. It is expected that this study will provide valuable information and direction for the development of effective treatment to GC in the future.

## 2 The origin of GCSCs

**Figure 1:**
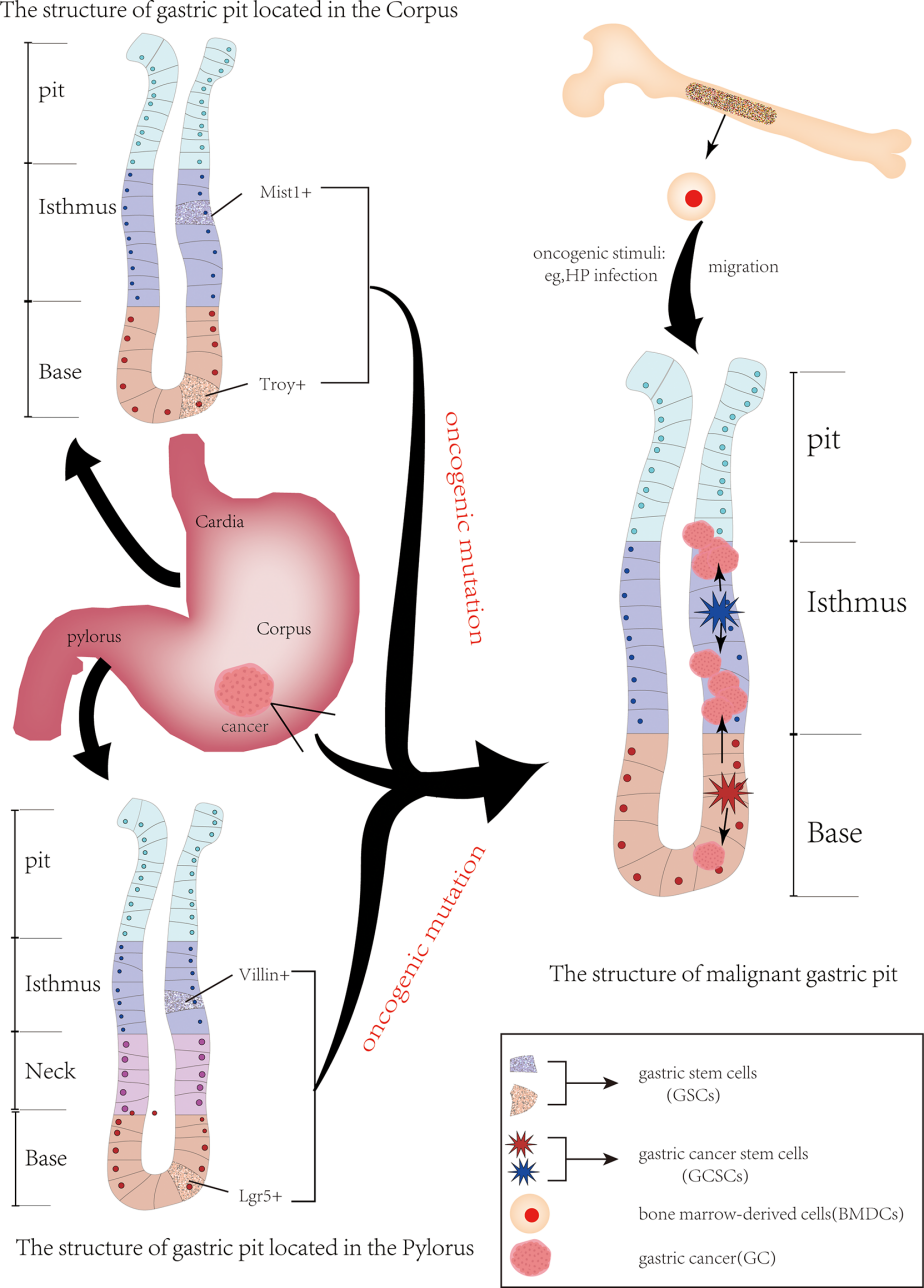
The origin of gastric cancer stem cells. The two primary sources of gastric cancer stem cells (GCSCs) are gastric stem cells (GSCs) and bone marrow-derived cells (BMDCs). Following carcinogenic mutation GSCs are converted into GCSCs. When stomach suffer oncogenic stimulation (e. g. HP infection), BMDCs, including bone marrow-derived mesenchymal stem cell cells, are recruited in the damaged area of the gastric mucosa, potentially serving as a source of GCSCs.

Since CSCs were first identified in acute myeloid leukemia and then in numerous other cancers, they have gradually become a hot topic in the field of cancer research (18). In 2009, CSCs were identified for the first time in human GC cells by using the cell surface marker CD44 (19). Many studies on identifying GCSCs from GC by using other cell surface markers, such as aldehyde dehydrogenase (ALDH), CD133, CD166 and C-X-C chemokine receptor type 4 (CXCR4), have been reported (20–22). These studies demonstrate that GCSCs do exist in GC. In addition, knowing the origin of GCSCs will be helpful to understand the role of GCSCs in gastric carcinogenesis and its mechanism. However, the exact source of GCSCs is still unknown. Currently, the main sources of GCSCs include mutant gastric stem cells (GSCs) and bone marrow-derived cells (BMDCs) (**Figure 1**).

GSCs are adult pluripotent stem cells that exist in gastric tissue and can differentiate into various types of gastric mucosal cells (23). At present, GSCs are mainly located in the isthmus and bottom of the gastric pit, and they form various glands and mucosal epithelial cells of the stomach by differentiation into progenitor cells. Some of these newly formed progenitor cells can migrate to the upper portion of the glandular duct and gradually differentiate into pit cells, while others can move to the base of the gastric gland, differentiating and maturing into parietal cells, chief cells, mucus cells and endocrine cells, thus forming a complete gastric duct (24, 25). These GSCs were transformed into GCSCs after undergoing oncogenic mutation, resulting in atypical hyperplasia of the gastric mucosa and the formation of GC (26). The anatomy of the stomach is composed of the cardia, fundus, body of the stomach and pylorus, and GSCs exist in different anatomical regions of the stomach and express their specific markers.

Villin, first discovered in the pylorus, is a calcium-regulated actin-crosslinking protein. Qiao et al. (27) confirmed that although rare, Villin possessed great potential to differentiate into many types of gastric mucosal cells. Park et al. established the GC cell line NCC-S1 (S1) and its metastatic variant cell line NCC-S1M (S1M) from a Villin-cre (transgenic mice expressing Cre recombinase from the villin promoter). And S1M exhibit characteristics of CSCs, such as strong carcinogenicity and drug resistance. They identified stem cell antigen 1 (Sca-1) as a cell surface marker whose expression was significantly increased in S1M. Compared with the cells with low Sca-1 expression, the cells with high Sca-1 expression were more tumorigenic and resistant to cisplatin chemotherapy by activating the TGF-β pathway and inhibiting the Wnt pathway (28). In addition, mouse models of GC established from a Villin-Cre have been shown to possess properties of CSCs in other studies (29). Another gastric epithelial stem cell that appears in the pylorus is Lgr5+ cells (leucine-rich repeat-containing G protein-coupled receptor 5) located at the base of the gastric glands. Lgr5, also known as G protein-coupled receptor 49, was first identified by Aaron J. W. Dr Hsueh’s team, and the overexpression of Lgr5 has been reported in a variety of cancers, such as breast cancer (30). And it has been shown to be the origin of CSCs. When stimulated by carcinogenic damage, these Lgr5-marked gastric epithelial stem cells become the source of GCSCs and GC (31–33). The Troy+ chief cells located at the base of the gastric corpus are thought to have the potential to differentiate into all gastric epithelium. When the tissue is stimulated by external injury, the Troy+ chief cells are able to act as gastric epithelial stem cells to differentiate into gastric cells that need to be repaired. When stimulated by carcinogens, Troy+ chief cells may become a source of GCSCs and promote gastric cancer formation (24, 34). Similarly, mist1+ GSCs located at the isthmus of the corpus gland are also considered the origin of all gastric epithelial cells. Generally, more than 98% of mist1+ isthmus stem cells are quiescent, while only 1.1% of mist1+ stem cells are Ki67+. When Kras mutation occurs, the percentage of Ki67+ mist1+ cells in the mist1+ isthmus stem cells increases so that these mist1+ isthmus stem cells attain the properties of CSCs. This in turn leads to intestinal metaplasia (IM) and dysplasia of the gastric epithelium. Thus, mist1+ isthmus stem cells are thought to be the origin of Kras-induced IM and hypoplasia. Further studies confirmed that they can serve as a cellular origin for all histologic types of GC, including intragastric-type and diffuse-type (35).

BMDCs have been considered as another possible source of GCSCs. BMDCs possess strong properties of plasticity and mobility. When stimulated by infection or injury, BMDCs are recruited to the site of infection or tissue damage to repair following inflammatory damage (36). In the meanwhile, some BMDCs are also recruited into the progenitor cell region, leading to gastric metaplasia and heterosis (the characteristics of CSCs) (37). Varon et al. showed that mice infected with different strains of HP developed intestinal metaplasia and dysplasia accompanied by the recruitment and accumulation of BMDCs in the gastric epithelial mucosa. And nearly 25% of the dysplastic lesions contained BMDC-derived cells (38). In addition, bone marrow-derived mesenchymal stem cells (BM-MSCs) are also considered to be involved in the occurrence and progression of HP-associated GC. They have been confirmed to be involved in the progression of GC by secreting thrombospondin-2 (39) and upregulating c-Myc (40). And BM-MSCs can also induce the formation of new tumor blood vessels in HP infection-associated GC by regulating the THBS4/integrin α2 axis and activating the PI3K/AKT pathway in endothelial cells (41). These findings suggest that BMDCs may be a source of GCSCs. However, more rigorous and adequate evidence is needed to support this conclusion.

## 3 The effect of GCSCs on GC

In recent years, CSCs have become a vital research area in tumor biology. CSCs are considered to be a small subset of tumor cells with the capacity for self-renewal, unlimited proliferation and relative dormancy and are thought to be involved in tumor progression, immune escape, recurrence, metastasis, and drug resistance. A growing body of research confirms the existence of GCSCs and their role in GC progression, immune escape, recurrence and metastasis (42). Han et al. isolated and cultured GCSCs and CAFs from resected GC specimens obtained from GC patients. They found that NRG1 secreted by CAFs promoted GC progression through modulating the self-renewal of GCSCs (43). Yes-associated protein (YAP) is involved in the progression of many malignancies and is thought to enhance the expression of GCSCs surface markers and self-renewal GCSCs *via* TGF-β-activated kinase 1 (44) or by inhibiting the expression of lipocalin-type prostaglandin D2 synthase (L-PTGDS) and prostaglandin D2 receptor 2 (PTGDR2) (45).And the increased expression of GCSCs markers and renewal of GCSCs will in turn promote the progression of GC and the self-renewal of GC cells (45).

### 3.1 GCSCs and the immune evasion of GC

In the initial period of tumor development, the human immune system is able to effectively identify, attack and destroy tumor cells. However, an increasing number of studies suggest that CSCs (including GCSCs) can effectively evade the surveillance of the immune system and its killing effect through different mechanisms (42, 46, 47). There is a close interaction between GCSCs and the immune system. On the one hand, GCSCs can contribute to giving rise to an immunosuppressive tumor microenvironment to avoid immune surveillance and killing; on the other hand, the immune cells surrounding GCSCs can convert non-GCSCs into GCSCs and maintain their properties of CSCs.

The number of tumor-infiltrating macrophages is closely related to the progression and prognosis of the tumor, and these tumor-infiltrating macrophages are named tumor-associated macrophages (TAMs). In GC, TAMs can be divided into the immunosuppressive M2 phenotype (TAMs-2) and the proinflammatory M1 phenotype (TAMs-1). And the former shows anti-inflammatory properties associated with the progression of tumors, while the latter exhibits proinflammatory and antitumor properties associated with the suppression of tumors (48). GCSCs can influence the immune microenvironment of GC by recruiting TAMs, while TAMs, in turn, can play a key role in maintaining CSC characteristics. Li et al. reported that GC mesenchymal stem cells were able to induce TAMs polarization into a TAMs-2 phenotype through the secretion of IL-6 and IL-8 by the JAK2/STAT3 signaling pathway. Meanwhile, these TAMs-2 can promote the epithelial-mesenchymal transition (EMT) process of GC, which in turn increases the migration and invasiveness of GC (49). In a study exploring the role of GCSCs markers in the prognosis and immunologic infiltration of GC patients, Lin et al. found that the overexpression of CXCR4 (a GCSCs marker) was related to the poor prognosis of GC patients, while EPCAM and TFRC (two GCSCs marker) were positively associated with the prognosis of GC patients. These GCSCs markers were associated with the infiltration and activation of different tumor-infiltrating immune cells, such as TAMs, of which CXCR4 was strongly positively correlated with TAMs. Therefore, GCSCs markers may maintain their tumor stem cell-like properties by recruiting distinct tumor-infiltrating immune cells such as TAMs in the tumor microenvironment (50).

Moreover, some studies (51) (52)have shown that cancer-associated fibroblasts (CAFs) play an important role in maintaining and enhancing the capabilities of CSCs in GCSCs. In a study, Hasegawa et al. attempted to clarify the effects of CAFs on the properties of CSCs in GC by using scirrhous gastric cancer cell lines (OCUM-12 and OCUM-2MD3) and non-scirrhous gastric cancer cell lines (MKN-45 and MKN-74). They found that CAFs may be able to maintain and enhance the tumor stem cell-like properties of GCSCs by modulating the TGF-β signaling pathway (51). Maeda et al. established 12 pairs of gastric CAFs and their corresponding non-CAFs from surgical specimens of GC and conducted genome-wide DNA methylation and H3K27me3 analyses in these specimens. The results of this study showed that H3K27me3 is responsible for the tumor-promoting ability of CAFs. Deletion of H3K27me3 enables CAFs to secrete some multiple stem cell niche factors, including WNT5A, GREM1, NOG and IGF2. Among these factors, WNT5A may promote the invasive ability of GCSCs. And inhibition of WNT5A secreted by CAFs can further inhibit GC cell proliferation and migration (52).

Immune checkpoint inhibitors have become a hot spot in the field of tumor therapy. The immune checkpoint is an important mechanism to prevent the immune system from attacking the body’s normal cells. Tumor cells also use this mechanism to evade immune surveillance and to suppress the immune attacks of T cells. At present, cytotoxic T lymphocyte antigen 4 (CTLA4) and programmed cell death 1 (PD-1)/PD1 ligand 1 (PD-L1) inhibitors are the main immune checkpoint inhibitors on the market (53). For example, B7-H1 is a ligand of PD-1 conventionally believed to convey inhibitory signals to T cells to suppress immune responses. Yang et al. reported that compared with B7-H1- GCSCs, B7-H1+ GCSCs showed stronger proliferative ability. Further study showed that stimulation of B7-H1 increased the level of Ki67 in GCSCs of LGR5+/B7-H1+, which in turn increased the ability of proliferate in B7-H1+/Lgr5+ GCSCs (54). However, the number of studies on the interaction between immune checkpoint inhibitors and GCSCs is relatively small.

### 3.2 GCSCs and drug resistance, relapse and metastasis of GC

**Figure 2:**
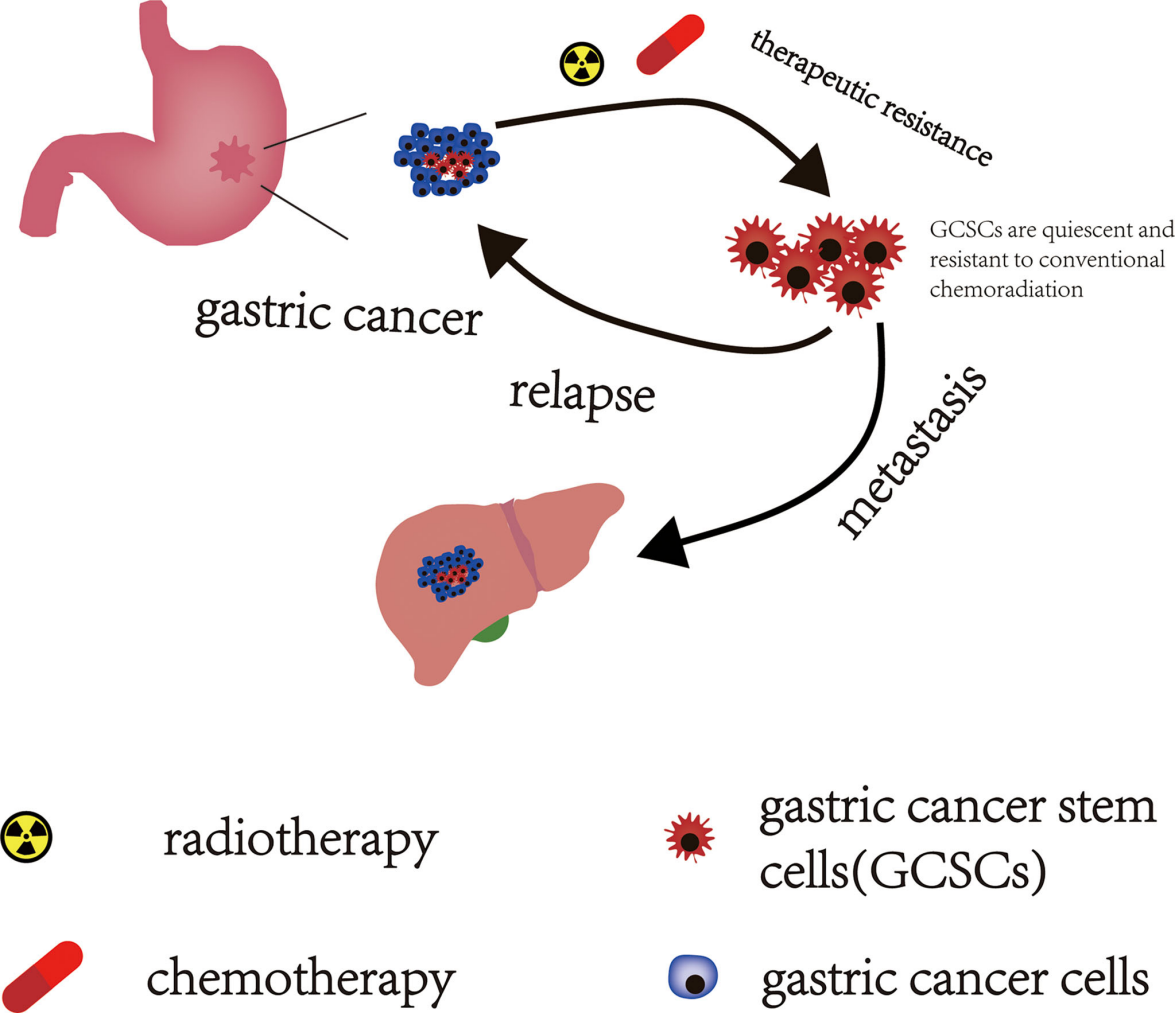
The effect of GCSCs on GC. GCSCs in a “dormant state” can escape the lethal effects of conventional chemoradiotherapy and survive after chemoradiotherapy. These surviving GCSCs act as “seeds” that drive GC recurrence and metastasis.

Drug resistance is a major obstacle in the treatment of GC and a key reason for the poor prognosis of GC. In recent years, increasing evidence suggests that GCSCs are involved in the formation of drug resistance in GC patients (**Figure 2**). Zhang et al. demonstrated that PIN1 suppressed chemotherapy resistance of GC cells by targeting GCSCs and multiple signaling molecules and biomarkers in GC (55). Yoon et al. found that the RhoA signal is able to promote and maintain the CSCs phenotypes in Lauren diffuse type gastric adenocarcinoma (DGA) cells. The activity of RhoA was negatively correlated with overall survival in DGA patients. Furthermore, inhibiting the RhoA signaling pathway can reverse the resistance to chemotherapy in DGA mouse xenograft models and in DGA CSCs (56). And another signaling pathway, RAC1 activity, also plays an important role in maintaining the CSCs phenotype of gastric adenocarcinoma (GA). Similarly, targeted inhibition of the RAC1 pathway also prevented EMT and CSCs phenotypes of GA, which in turn inhibited the progression, metastasis, and drug resistance of GA (57).

Relapse and metastasis of patients with GC following combined modality therapy are primary poor prognostic factors in patients with GC. CSCs underlie the development and progression of cancer and are also responsible for driving tumor recurrence and metastasis (**Figure 2**). The STAT3 signaling pathway is thought to promote the acquisition of tumor stem cell-like characteristics and EMT in cancer (58). Jiang et al. reported that IL-17 fosters more aggressive development of GCSCs by promoting the activation of its downstream STAT3 transcription factor pathway, and invasive GCSCs are closely related to the occurrence and metastasis of GC by promoting EMT. Stattic (a STAT3 specific inhibitor) can significantly reduce the invasiveness of GCSCs, thereby inhibiting the occurrence and metastasis of GC (59). Yoon et al. explored the effect of KRAS on the EMT and CSCs phenotype of GA in patient-derived GA cell lines (AGS and KATOIII) and in a mouse model of GA with loss of p53 and Cdh1 that added oncogenic KRAS (a.k.a. Tcon mice). They found that activation of KRAS signaling could promote the EMT of GA cells and enable GA to acquire the phenotype of CSCs, both in Tcon mice and in cell lines of AGS and KATOIII, which then enhanced the invasive and metastatic potential of GA. And inhibiting or knocking down KRAS could reduce the EMT and CSCS phenotypes of GA, which in turn attenuated the invasiveness of GA and reduced the occurrence of experimental pulmonary metastasis (60).

## 4 Potential therapeutic strategies for GC: Targeting GCSCs

**Figure 3:**
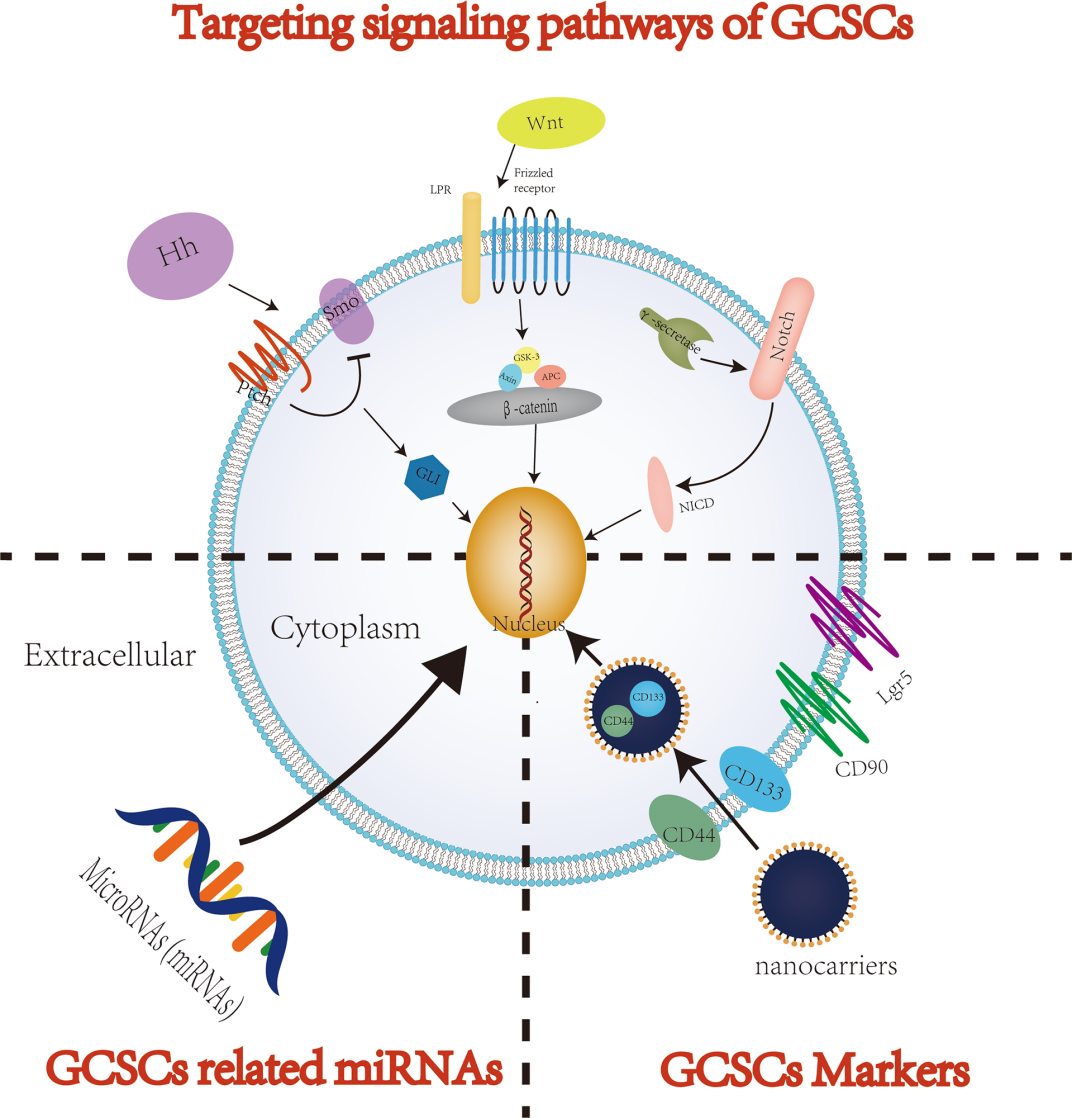
The potential therapeutic strategies targeting GCSCs.

In recent years, despite significant progress in therapy of GC, the overall effects of the available systemic therapies for advanced GC patients remain limited. An increasing number of studies have shown that when used to treat tumors, including GC, traditional antitumor treatments, such as chemoradiotherapy, usually attack and kill rapidly proliferating cancer cells (61). However, CSCs, including GCSCs, are considered as tumor cells initiating cells and are usually in a quiescent/dormant state. Therefore, the dormant CSCs can survive anti-tumor therapy and be restored to rapidly proliferating tumor cells through cellular plasticity, thereby eventually resulting in recurrence and metastasis after tumor treatment (62). In addition, conventional tumor therapies suffer from lacking specificity to tumors, which may cause serious damage to normal tissues, further reducing the quality of life of patients with GC. In order to completely eliminate all GC cells and prevent recurrence and metastasis after treatment, it is crucial to specifically kill GCSCs responsible for tumor initiation, progression, recurrence, metastasis and drug resistance. Therefore, targeted elimination of GCSCs, which lead to cancer cell growth and maintain their progression, is considered to be one of the most promising solutions to improve the treatment efficiency of GC patients. With the deepening of research on GCSCs, the current methods of targeted therapy for eradicating GCSCs mainly focus on three methods: targeting cell surface markers, signaling pathways and microRNAs (miRNAs) (**Figure 3**). However, methodological dilemmas also exist for the current targeted therapy of eradicating GCSCs, such as methods to specifically identify and isolate GCSCs. This article summarizes the existing research on the specific methods of targeted therapy for GCSCs, and this information may provide theoretical support for the future development of highly specific therapeutics targeting GCSCs. **Table 1** summarizes the key GCSCs-related cell surface markers and signal pathways.

**Table 1:** The role and regulation of GCSCs-related cell surface markers and signal pathways in GC.

Drug/Molecule	Cell surface markers/Signal pathways	Effect on Signal pathways	Major outcomes	Refs
–	CD44+,CD44v,Combined CD44+/CD54+,Combined EpCAM+/CD44+	Wnt/β-catenin pathway	Higher tumorigenicity and Spheroid formation	(19, 63–65)
–	Combined CD44+/CD24+	Notch pathway	Higher tumorigenicity	(66)
–	CD71-	–	have high tumorigenicity, multipotency, and invasiveness abilities	(67)
–	CD133	–	Promote the expression of ki-67 of GC cells	(68)
–	Lgr5	–	Play a role in the development and progression of GC	(69)
–	ALDH1+	–	Showe higher tumorigenic potential, self-renewal, and produce heterogeneous cell populations	(70)
ICG-001, RNF43, RORβ, Ibuprofen	Wnt/β-catenin	Down	Inhibit GCSCs characteristics, the growth, chemoresistance and metastasis of GC cells	(71–74)
NANOGP8, Frizzled 7	Wnt/β-catenin	Up	Enhance proliferation, migration, invasion, sphere-forming and clonogenic capacity, and chemoresistance of GC cells	(75, 76)
RO4929097(a selective inhibitor of γ-secretase)	Notch	Down	SD in 6 of 33 evaluable metastatic CRC patients (18.2%)	(77)
GSI	Notch and Wnt/β-catenin	Down	Inhibit the aggressiveness and tumorigenicity of GC	(78)
Vismodegib	Hh	Down	does not promote the efficacy of standard therapy for metastatic CRC	(79)
sulforaphane	Hh	Down	Inhibit the proliferation of GCSCs	(80)
apatinib	SHh	Down	Inhibit tumor formation, cell proliferation and the expression of GCSCs markers	(81)
PTPRU	Hippo/YAP	Down	Inhibit the stemness of GCSCs	(82)
SCD1	Hippo/YAP	–	Promote GCSC stemness	(83)

### 4.1 Targeting surface markers of GCSCs

One strategy to eradicate GCSCs mainly focuses on targeting GCSCs specific surface markers. CD44 was the first cell surface marker identified as a potential GCSCs-specific cell surface marker (47). Studies have shown that CD44, CD133 and CD90 are GCSCs markers and are associated with properties of GCSCs, such as the capacity of self-renewal and promoting tumor progression (84–88). Gamma-secretase inhibitor IX (GSI) was confirmed to effectively inhibit the proliferation, migration and invasiveness of GC cells and induce apoptosis of GC cells by targeting CD44+ GCSCs. Barat et al. further found that GSI concomitantly inhibited the Notch and Wnt/β-catenin signaling pathways by specifically targeting CD44+ GCSCs, thereby suppressing the progression of GC (78). Using three complementary models, including 2D and 3D *in vitro* culture systems as well as tumor xenografts in mice, Nguyen et al. sought to explore the interaction between all-trans-retinoic acid (ATRA) and GCSCs and its mechanism. And they found that ATRA was able to inhibit the self-renewal and tumorigenic properties of GCSCs by targeting GCSCs markers such as CD44 and ALDH (89). Furthermore, Lgr5, an identification marker of CSCs, plays a critical role in tissue development and the maintenance of adult stem cells in the gastrointestinal tract. And Lgr5 is also considered as another potential marker of GCSCs and a downstream target in Wnt/β-catenin signaling that is responsible for the progression of GC (32, 90). Wang et al. found that Lgr5 was intimately associated with the stemness regulators and the EMT inducers of GCSCs (91). And Lgr5 promoted by regulatory T cells is also thought to confer poor prognosis in GC through TGF-β involved in activation of the Wnt signaling pathway (90).

Although targeting GCSCs with a surface marker may be a promising therapeutic strategy for eradicating GCSCs, some questions of traditional approaches to targeting CSCs remain to be addressed, including poor water solubility, poor pharmacokinetics, and poor stability of CSC-specific agents (92, 93). In fact, delivering drugs to CSCs, which account for a tiny fraction of tumor tissue, is a huge challenge. In recent years, drug delivery methods based on nanocarriers have offered the following advantages, including controlled drug release and improved biodistribution, providing an effective targeted therapy strategy for successfully targeting CSCs, including GCSCs (93). Studies have shown that targeting CSCs therapy based on nano-delivery systems was able to eliminate CSCs more efficiently with lower toxicity than targeting CSCs therapy without nanocarrier delivery (92–94). And this conclusion is also true for targeting GCSCs therapy. On the other hand, the accurate delivery of nanomedicine to GCSCs by targeting the surface markers of GCSCs is also a promising therapeutic strategy. Chen et al. successfully delivered nanoparticles to CD133+ and CD44+ GCSCs by targeting CD133 and CD44 with CD133 and CD44 antibodies. Compared with single targeting (targeting CD44 or CD133) and without targeting GCSCs markers, nanomedicines targeting both CD133 and CD44 can inhibit the growth of GCSCs more effectively (87). As a member of the ubiquitin-specific proteases (USPs), USP22 is considered to be related to tumor invasion and metastasis, and also participates in maintaining the characteristics of CSCs. Yang et al. developed a drug delivery system, USP22-NLs-CD44, to enhance the targeted therapy for GSCCS of USP22 siRNA by combining CD44 antibody with USP22 siRNA-loaded nanoliposomes. USP22-NLs-CD44 was confirmed to be able to specifically deliver USP22 siRNA to CD44+ GCSCs, and it could eradicate CD44(+) GCSCs more effectively than nanoliposomes without targeting CD44 (95). Yao et al. developed a novel therapeutic siRNA nanoparticle by targeting glioma-associated oncogene homolog 1 (Gli1) and CD44 of GCSCs. And this siRNA nanoparticle exhibits significant inhibition of the characteristics of GCSCs *via* specifically downregulating Hedgehog (Hh) signaling both *in vitro* and *in vivo (* 17).

### 4.2 Targeting signaling pathways of GCSCs

Signaling pathways (e.g., Notch, Hedgehog, Wnt/β-catenin) are essential for maintaining normal stem cell physiology and self-renewal. The abnormality or dysregulation of these signaling pathways is also a key factor in tumor occurrence, development and maintenance of tumor stem cell-like properties. Therefore, targeting these signaling pathways that regulate the biological properties of GCSCs may become a promising therapeutic strategy for targeting GCSCs.

#### 4.2.1 Wnt/β-catenin signaling pathway

Wnt/β-catenin signaling pathway is considered to be critical for tissue repair and stem cell renewal. Wnt/β-catenin signaling pathways are generally categorized into canonical and non-canonical signaling pathways according to whether β-catenin is activated (96). In the process of carcinogenesis, the canonical Wnt/β-catenin pathway participates in the maintenance and proliferation of GCSCs, while the non-canonical Wnt/β-catenin pathway is associated with EMT and the initiation of GCSCs (97). Liu et al. showed that ICG-001 significantly inhibited the stem cell-like properties of GCSCs and the growth, chemoresistance and metastasis of GC cell lines by blocking the Wnt/β-catenin signaling pathway (71). DOCK6, a guanine nucleotide exchange factor (GEF) for Rac1 and CDC42, has been shown to be capable of promoting the stemness of GCSCs by modulating the Wnt/β-catenin signaling pathway. While the stemness of GCSCs in turn promotes the progression, chemoresistance and radioresistance of GC (98). Conversely, as a member of the transmembrane E3 ubiquitin ligase family, ring finger protein 43 (RNF43) has been shown to weaken the stemness of GCSCs by regulating the Wnt/β-catenin signaling pathway. And RNF43 was also confirmed to be related to the progression of GC. While this trend can be reversed to some extent by activating the Wnt/β-catenin signaling pathway through the Wnt pathway activator (72). In the study of the effect of knockdown of Placental growth factor (PIGF) on cell apoptosis in GCSCs, Akrami et al. found that PIGF can induce apoptosis in GCSCs *via* influencing the Wnt signaling pathway (99). Wen et al. reported that retinoic acid−related orphan receptor β (RORβ) inhabited the ability of tumor formation and the stemness of GCSCs by suppressing the Wnt/β−catenin signaling pathway activity *in vivo*. In addition, overexpression of RORβ attenuated the activity of GC cells and induce the apoptosis of GC cells *via* upregulation of Bcl−2 like protein 11 (a pro−apoptotic gene) (73). Ibuprofen was demonstrated by Akrami et al. that it might diminish the proliferation of GC cells and attenuate the CSCs properties of GCSCs by blocking the Wnt/β−catenin signaling pathway (74). According to Jung et al., human epidermal growth factor 2 (HER2), which has a significant influence on breast CSCs, has also been found to affect GCSC activity *via* modulating the Wnt/-catenin signaling pathway. And GC cells with HER2 overexpression were intimately associated with the stemness and invasion of GCSCs by regulating the Wnt/β-catenin signaling pathway (100). Ma et al. found that NANOGP8 was not only a key regulator of maintaining the CSCs properties of GC cells and enhancing Wnt signal transduction, but was also closely related to EMT, chemoresistance and other malignant characteristics of GC cells (75). Frizzled 7 (FZD7), a member of the Frizzled family, is closely related to the proliferation and invasiveness of many tumors. In GC, the expression of FZD7 was upregulated and FZD7 promoted GC progression, including GC proliferation, invasion and migration. In 2018, Li et al. revealed a potential mechanism that FZD7 promotes GC progression. The expression of FZD7 significantly promotes the CSCs-like properties and EMT of GC by activating the canonical Wnt/β-catenin signaling pathway (76). HP infection is considered to be a major pathogenic factor of non-cardia GC. And Yong et al. found that HP infection-associated GC exhibited CSCs properties, such as increased expression of Nanog and Oct4 (two CSCs specific surface markers). Further research demonstrated that HP-induced upregulation of Nanog and Oct4 increased the CSCs properties of GC *via* the Wnt/-catenin signaling pathway (101).

#### 4.2.2 Notch signaling pathway

The Notch signaling pathway is critical for the maintenance of the cancer stem or progenitor cell compartment required for tumorigenesis in GC. Notch signaling is one of the most activated pathways in gastric tumorigenesis and plays a critical role in the links between gastric carcinogenesis and gastric stem (progenitor) cell proliferation (102). Barat et al. reported that GSI-mediated targeted therapy of CD44+ GCSCs exhibited inhibitory effects on the aggressiveness and tumorigenicity of GC by suppressing the Notch and Wnt/β-catenin signaling pathways (78). Autophagy is thought to be involved in tumor progression. A growing body of evidence suggests that autophagy can assist tumors in dealing with external pressures, such as hypoxia, nutrient shortage or cancer treatment, and then promote tumor progression (103, 104). Similarly, under the environment of external stress, GCSCs have also been confirmed to sustain their cell activity through the autophagy system (105). Therefore, autophagy inhibitors are also considered as one of the potential therapeutic agents for cancer including GC. And the maintenance effect of autophagy on the cell viability of GCSCs is related to the abnormality of Notch signaling pathway. Li et al. reported that chloroquine, an autophagy inhibitor, exhibited an inhibitory effect on the cell viability of GCSCs, and its inhibitory effect was significantly enhanced when combined with 5-fluorouracil. Further studies showed that autophagy can regulate the drug resistance of GCSCs by regulating the Notch signaling pathway (106).

#### 4.2.3 Hedgehog signaling pathway

The Hh signaling pathway plays a crucial role in embryonic development and maintenance of tissue homeostasis, and it is also involved in the self-renewal and stemness maintenance of CSCs (107). In addition, the Hh signaling pathway has also been considered as a key pathway to maintain the stemness of GCSCs (80). Sulforaphane, an extract of broccoli/broccoli sprouts, has been shown to possess antitumor activity. Ge et al. found that sulforaphane inhibited the proliferation of GCSCs and induced their apoptosis by inhibiting the activity of the Hh signaling pathway (80). Dysregulation of the sonic Hh (SHh) signaling pathway, a type of Hh signaling pathway, plays an important role in maintaining the properties of GCSCs. Apatinib, an oral small-molecule tyrosine kinase inhibitor, has been used to treat advanced GC in China. Cao et al. found that apatinib inhibited tumor formation, cell proliferation and the expression of GCSCs markers. Moreover, it also significantly inhibited tumor growth and the CSCs properties of GCSCs in a xenograft tumor model. The anti-tumor and anti-GCSCs properties of apatinib were mediated by drastically lowering the protein expression of SHh pathway molecules, such as family zinc finger (GLI1) and GLI2. And the use of paramorphine, an activator of the SHh signaling pathway, enhanced GC progression and the CSCs properties of GCSCs. When apatinib was combined with paramorphine, the inhibitory effect of apatinib on tumor growth and GCSCs properties would be attenuated (81). As a transcription factors of the Hh signaling pathway, aberrant expression of GLI2 has been associated with a variety of malignancies. Lu et al. found that high expression of miR-144-3p significantly inhibited the proliferation and invasiveness of GC and stemness of GCSCs. This inhibitory effect was also observed in xenografted tumor mice. It was also discovered using tumorsphere formation assays and flow cytometry that the inhibitory impact of miR-144-3p on GC development and GCSC stemness was achieved through controlling the expression of GLI2 (108).

#### 4.2.4 Other potential signaling pathways

Similar to Wnt/β-catenin, Hippo/YAP is a highly conserved signal transduction pathway that contributes to the regulation of self-renewal and differentiation of CSCs while also being implicated in cancer development and maintenance. As a signaling regulator that modulates various cellular processes, PTPRU was shown to attenuate the stemness of GCSCs by inhibiting the expression of YAP and thereby inhibiting the Hippo/YAP signaling pathway. And PTRPU knockdown strengthens the stemness of GCSCs (82). Stearoyl-CoA desaturase-1 (SCD1) is thought to be responsible for the stem cell-like properties of tumors. Similarly, Gao et al. reported that SCD1 increased the stem cell-like properties of GCSCs through the Hippo/YAP signaling pathway, which is a key “seed” for GC tumorigenesis, chemoresistance, and metastasis. Conversely, inhibition or knockdown of SCD1 by siRNA attenuated the stem cell-like properties of GCSCs (83). By using GC cell lines (SGC-7901, MKN-45) to study the effect of GLI2 on the expression of PDGFRB and the effect of Gli2 and PDGFRB on the characteristics of GCSCs, Wang et al. found that GLI2 evaluated the expression of PDGFRB at both the mRNA and protein levels. Additionally, knocking down either Gli2 or PDGFRB lowered the expression of CSCs related genes, such as CD44, in GC cells. Therefore, the GLI2-PDGFRB axis may be a potential signaling pathway for targeted therapy of GCSCs (109). FOXO1 is a transcription factor closely related to the progression and metastasis of GC. Choi et al. reported that FOXO1 could inhibit the tumorigenic ability of GC cells and regulate the stemness of GC cells by interacting with the GCSCs marker Lgr5. Thus, they believe that the FOXO1/Lgr5 signaling pathway may be a potential target for GC therapy by modulating the stemness of GCSCs (110). Ran et al. reported another signaling pathway, the JNK signaling pathway, related to the CSCs properties of GCSCs. They found that GREM2 exhibited great potential to regulate the proliferation, migration, invasiveness and apoptosis of GCSCs by regulating the JNK signaling pathway. Inhibition of the GREM2 or JNK signaling pathway not only suppresses the proliferation and invasiveness of GCSCs and promotes the apoptosis of GCSCs *in vitro* but also suppresses the tumor formation and lymph node metastasis of GCSCs *in vivo (* 111). Zhang et al. found that SLC34A2, as a key regulator of miR-25 transcription, could regulate the tumorigenesis and self-renewal properties of CD44+ GCSCs. And they also showed that miR-25 directly targets Gsk3β in CD44+ GCSCs by luciferase assays. Therefore, they believe that the SLC34A2-miR-25-Gsk3β pathway is a possible pathway regulating the properties of GCSCs and GC progression and is expected to be a potential target for the treatment of GC (112).

In conclusion, GCSCs-related signaling pathways may become potential therapeutic targets for the eradication of GCSCs in the future. However, one point to note is that the current research on targeting GCSCs for GC therapy *via* GCSCs-related signaling pathways is almost all preclinical theoretical research. Therefore, the application of this therapeutic strategy in the treatment of GC still has a long way to go.

### 4.3 MicroRNAs (miRNAs)

MicroRNAs (miRNAs) may be important modulators of the characteristics of GCSCs. For instance, miR-598 was shown to inhibit the growth and invasiveness of GC cells by attenuating the self-renewal properties of GCSCs (113). CD44 is an important surface marker of GCSCs, Lee et al. found that miR-193a-3p was overexpressed by/CD44(+) GC cells compared with CD44(-) GC cells. And the expression of SRSF2, the target gene of miR-193a-3p, was downregulated in CD44(+) GC cells. Further research revealed that the expression of anti-apoptotic genes such as Bcl2 and Bcl212 was upregulated, whereas pro-apoptosis genes such as Bax and cytochrome c were downregulated in CD44(+) GC cells. Furthermore, increasing the expression level of miR-193a-3p stimulated the progression of cisplatin resistance in CD44(+) cells of GC patients. Inhibition of miR-193a-3p expression increases the expression of SRSF2 and alters the expression level of multiple apoptosis-related genes, which further reduces cell activity and increases cell apoptosis in CD44(+) cells (86). Pan et al. found that the expression of miR−196a−5p was significantly upregulated in CD44(+) GC cells compared to CD44(-) cells by miRNA microarray analysis. MiR−196a−5p inhibited the expression of Smad4, which was positively related to the TNM stage and aggressiveness of GC. And overexpression of Smad4 also significantly suppressed EMT stimulated by miR−196a−5p in GCSCs. Therefore, miR−196a−5p may play a significant role in the EMT and invasiveness of GCSCs by targeting Smad4 (114). Zhao et al. reported that miR-6778-5p could positively regulate SHMT1 expression by targeting YWHAE in Drosha-silenced GC cells. And SHMT1 plays a crucial role in maintaining the CSCs properties of GCSCs by regulating cytosolic one-carbon folate metabolism. Furthermore, targeted inhibition of miR-6778-5p or SHMT1 attenuated GCSC spherical formation and increased therapeutic sensitivity to 5-fluorouracil in Drosha-knockdown GC cells (115). MiR-375 has been shown to reduce the stemness of GC cells *in vitro* and *in vivo* by directly targeting SLC7A11, which could trigger SLC7A11-dependent ferroptosis (116). Studies have shown that miRNAs may affect the properties of GCSCs by regulating GCSCs-related signaling pathways. Xin et al. showed that elevated expression of miR-7-5p reduced the invasiveness of GCSCs by targeting Smo and Hes1 and subsequently blocking Notch and Hedgehog signaling pathways *in vitro*. Likewise, upregulation of miR-7-5p expression also suppressed GC growth in a xenogeneic model (117). Shao et al. reported that miRNA-19b/20a/92a promoted the self-renewal of GCSCs by activating the Wnt/β-catenin signal transduction pathway. Similarly, Fan et al. found that miR-501-5p could significantly promote the CSC properties of GC cells by activating the Wnt/β-catenin signaling pathway *via* targeting DKK1, NKD1 and GSK3β (118). In addition to regulating the stemness of GCSCs, miRNAs can also affect the drug resistance of GC cells. Zhan et al. demonstrated that miR-98 not only inhibited the stemness of GCSCs but also increased the sensitivity of GC cells to cisplatin treatment by targeting branched-chain aminotransferase 1 (119). Similarly, the increased expression of miR-132 in Lgr5+ GCSCs has been shown to contribute to cisplatin resistance of GC by regulating the SIRT1/CREB/ABCG2 signaling pathway (120). Taken together, these studies suggest that miRNAs may be promising potential targets for targeted therapy of GCSCs (121).

### 4.4 LncRNAs

As another class of non-coding RNA, many lncRNAs have been confirmed to be dysregulated in GCSCs. Recent research sheds light on the crucial roles that lncRNAs play in regulating cell proliferation, drug resistance, interaction with key signaling pathways, and GCSC-associated gene expression (122, 123). Sun et al., found that lncRNA LOXL1-AS1 was overexpressed in tissues and cells of GC and the upregulation of LOXL1-AS1 was associated with poor prognosis in GC. Their results showed that LOXL1-AS1 induced cell proliferation, migration, EMT, and stemness, which greatly facilitated the progression of GC. Additionally, the expression of USF1 was higher in GC than in healthy controls and LOXL1-AS1 worked as a ceRNA to upregulate USF1 by sponging miR-708-5p. They further verified that LOXL1-AS1 promote carcinogenesis and stemness in GC by regulating miR-708-5p/USF1 pathway (124). Similarly, Song et al., demonstrated that lncRNA THOR facilitates the stemness of GC cells by improving the stability of SOX9 mRNA (125). A lncRNA called ADAMTS9-AS2 plays a role in the genesis and progression of several malignancies, including GC. By combining bioinformatics analysis, Wang et al., discovered that ADAMTS9-AS2 positively linked with the expression of SPOP in GC tissues. Additionally, they revealed that ADAMTS9-AS2 hinder the progression of GC and sphere-forming capability by modulating SPOP expression (126). LncRNAs have also been confirmed to be involved in the process of MSCs promoting the development of drug resistance in gastric cancer. He et al. revealed that MSCs induced lncRNA MACC1-AS1 expression in GC cells *via* secretion of TGF-β1, while MACC1-AS1 enhanced FAO-dependent stemness and chemoresistance of GC cells through inhibiting miR-145-5p. Furthermore, *in vivo* MSC-induced FOLFOX treatment resistance was reduced by pharmacological FAO inhibition with etomoxir (ETX) (127). Likewise, lncRNA HCP5 induced by MSCs was reported to trigger FAO *via* miR-3619-5p/AMPK/PGC1/CEBPB axis boost stemness and chemo-resistance of GC, showing that inhibiting HCP5 was a novel way to improving the efficacy of chemotherapy in GC (128). These results contributed to our understanding of crosstalk between LncRNAs and GCSCs and may introduce a novel therapeutic target for GC therapy.

## 5 Discussion

### 5.1 Challenges and barriers of GCSCs-targeted therapies

Despite a growing body of research on targeting GCSCs for the treatment of GC, there are still some nonnegligible challenges and obstacles in targeting GCSCs for the therapy of GC.

Firstly, accurate identification and isolation of GCSCs is crucial for conducting GCSCs-related research. The incorrect identification of CSC subpopulations sometimes leads to incorrect findings. However, a major technical challenge in the field is to specifically identify and isolate GCSCs. It has been reported that CSCs are extremely rare in tumor cells, accounting for <1% of tumor cells (129). This makes it difficult to identify and isolate CSCs. At present, the primary strategy for identifying and isolating GCSCs is to use cell surface markers of GCSCs such as CD44 and CD133. And novel techniques such as single-cell sequencing methods may also help identify GCSCs (47). However, these methods cannot isolate GCSCs specifically. For example, some GCSCs do not express the cell surface markers found so far, whereas some non-GCSCs do express some GCSCs related surface markers (130).

Secondly, although dysregulation of many signaling pathways has been reported in GCSCs, these pathways are also critical to the maintenance of normal stem cell physiology. Therefore, agents targeting these signaling pathways may not only affect GCSCs, but also have some adverse effects on the normal physiological activities of stem cells. Therefore, more efforts should be made in the future to specifically target GCSCs without targeting normal stem cells.

Finally, the current therapeutic strategies targeting GCSCs are still in the preclinical phase of theoretical research. And most of these studies were performed *in vitro* or in animal models. Virtually no studies on GCSC-targeted therapy in GC have been reported. In-depth knowledge of the effective dose and side effects of this treatment should be well defined before it is applied to clinical practice. However, the current study does not speak to this point. In addition, the exact mechanisms that GCSCs participate in the progression, drug resistance, recurrence and metastasis of GC are still being explored.

### 5.2 Conclusion and future perspectives

In recent years, accumulating evidence suggests that GCSCs are the “seeds” that drive GC progression and play an important role in GC resistance, recurrence, and metastasis. Therefore, therapeutically targeting GCSCs represents a promising therapeutic strategy for the treatment of GC, ushering in a new era of GC therapy. Current studies suggest that targeting GCSCs-related markers, signaling pathways, and microRNAs (miRNAs) may be a promising therapeutic strategy to eliminate GCSCs. However, due to the intricate interactions between GCSCs and GCs, as well as the underlying mechanisms, more studies are still needed in the future to reveal the biological characteristics of GCSCs, such as the exact origin of GCSCs. In addition, there are still some unresolved puzzles in the field of GCSCs research. Future studies should focus on identifying specific GCSCs and in-depth knowledge of the pathogenic mechanisms of GCSCs in GC and thus deliver on its promise to revolutionize the therapy of GC. In summary, targeted therapy to eradicate GCSCs in combination with current antitumor therapy, including chemoradiotherapy and surgery, may be able to treat GC more thoroughly and provide a better prognosis in the future.

## Author contributions

Literature review, data analysis, and manuscript preparation were performed by YY. W-JM and Z-QW contributed for the study conception, design, and revision. All authors contributed to the article and approved the submitted version.

## Conflict of interest

The authors declare that the research was conducted in the absence of any commercial or financial relationships that could be construed as a potential conflict of interest.

## Publisher’s note

All claims expressed in this article are solely those of the authors and do not necessarily represent those of their affiliated organizations, or those of the publisher, the editors and the reviewers. Any product that may be evaluated in this article, or claim that may be made by its manufacturer, is not guaranteed or endorsed by the publisher.

## Glossary

**Table:**

ATRA	all-trans-retinoic acid
BMDCs	bone marrowderived cells
BM-MSCs	bone marrow-derived mesenchymal stem cells
CAFs	cancer-associated fibroblasts
CSCs	cancer stem cells
CXCR4	C-X-C chemokine receptor type 4
DGA	Lauren diffuse type gastric adenocarcinoma
FZD7	Frizzled 7
GA	gastric adenocarcinoma
GC	gastric cancer
GCSCs	gastric cancer stem cells
GEF	guanine nucleotide exchange factor
GLI	family zinc finger
Gli1	glioma-associated oncogene homolog 1
GSCs	gastric stem cells
HER2	human epidermal growth factor 2
Hh	Hedgehog
HP	Helicobacter pylori
Lgr5	leucine-rich repeat-containing G protein-coupled receptor 5
L-PTGDS	lipocalin-type prostaglandin D2 synthase
PlGF	Placental growth factor
PTGDR2	prostaglandin D2 receptor 2
RNF43	Ring finger protein 43
RORb	retinoic acid−related orphan receptor b
Sca-1	stem cell antigen 1
SCD1	Stearoyl-CoA desaturase-1
SHh	sonic Hh
TAMs	tumor-associated macrophages
USPs	ubiquitin-specific proteases
Villin-cre	transgenic mice expressing Cre recombinase from the villin promoter
YAP	Yes-associated protein
